# Supplementary material for: Redirecting patients from the pediatric emergency department to community locations for care: A qualitative study of healthcare professional and leader perspectives
Source: PLoS One. 2025 Dec 12;20(12):e0338725. doi: 10.1371/journal.pone.0338725 (PMC12700394; doi:10.1371/journal.pone.0338725)
Supplement: S1 File — (DOCX) [file pone.0338725.s001.docx]

## **Supporting Information File 1 - Additional Illustrative Quotes**

|  | **Illustrative Quote** | **Participant** |
| --- | --- | --- |
| Problem | I think so much of our wait times and the volumes we're seeing are kids who don't need to be seen in the ED, but it's mostly because there is a lack of primary care or lack of quick access to care for them so they end up in our EDs. | Pediatric ED Physician 2 |
|  | I would say 3/4 of patients I see, actually have a family doctor. The problem is, they can't get into them, and... since the pandemic, the number that only do video [appointments] is ridiculous. | Pediatric ED Physician 4 |
|  | I think we're even more short on family physicians than anything else. And if we were staffing a clinic like that, are we pulling a family physician from office practice? | Pediatric ED Physician 4 |
| Solution | Anything that we can do to decant from emergency, you know, to really operate as a true emergency department and better serve our community is so important. | Leader 7 |
|  | Very positive. I recognize the strains on the ED that are volume based and specialty based. So if there are opportunities to provide care to families closer to home at the appropriate level of care then I think that would be an important step for the system. | Leader 6 |
| System barriers | You would imagine the things that save the system money got people care better allowed people to be seen faster would be a win for everybody. But there are a lot of things that just are not on people's radar, or they don't have the time to do it. | Family Physician 1 |
|  | I feel like the politics involved are a huge barrier. Like they, you know, like the health authority does not want to fix upstream... I think that it is tough to get money for things that aren't so like demonstrative of the health authority making change to improve constituents’ lives. | Pediatric ED Physician 3 |
|  | There might actually be an increase in volume presentation to the emergency department seeking those appointments. | Pediatric ED Physician 5 |
| Confidence | I think everybody's everybody feels the primary care, sensitive conditions. But and would like to have somewhere to send them to, but have that bit of fear of what that actually looks like. So I think it just has to be very clear what each place offers to start and what everybody does. | Leader 4 |
|  | A lot more buy-in to this if they knew that there was capacity with community partners to see a child in a timely way. | Leader 6 |
|  | I think that you have to have everybody on the same page, and that we're all operating with the understanding of that we're going to be a bit more intersectional, because I think we often operate in silos between our acute services and our community services. So how do we have something bridging to ensure that we're each saying the same thing that we're offering the right services, that we understand each other. | Leader 4 |
|  | If you had one resuscitation after another, your 8 hour day would be quite a day at work. Whereas interspersing those, in a sense, those primary care visits, or those quick visits, sometimes gives you some time to kind of again de-stress between those other patients that are sicker. | Leader 1 |
| Evidence | Knowing that, like you know, other sites have done this successfully is often just like helpful to know. You know that similar centers have done it successfully, and that it's not just like something that we're trialing out of desperation or like just we made up out of out of thin air. | Triage Nurse 4 |
|  | There are ways to consider whether [the malpractice organization for physicians] ... made it clear that it's not a breach of duty ...I think that would go a long way, because at least on the physician side they rely very heavily on advice from [the malpractice organization for physicians] as to what risks they should and shouldn't take, and a lot of the questions focus on duty to care and breach, you know, withdraw of care and things like that. So those are pieces that I think would be important to explore in implementing a program that could mitigate this cultural fear and might lead to liberalization of pathways. | Leader 5 |
|  | It's really hard to be a triage nurse to begin with... if you're also a nurse who's then gonna offer/direct someone to other care, feeling like you're not gonna take on extra liability, feeling like you're supported by your leadership, and that you're cared for in those decisions, I think would be really important. | Pediatric ED Physician 1 |
| Training | [I would want] structured education and support with a program like this. Cause when we've had other things roll out, we've had no like official education on it...we had our inclusion exclusion, criteria but some of it we weren't really sure what the rationale was. | Triage Nurse 2 |
|  | Nurses are already having these kinds of conversations, and it would be, I think, a win to kind of pivot those conversations to ...provide some like nurse level reassurance of like their assessment, and then being able to say, "we have this program we have this opportunity for you"... I think, like we're already having, like the beginnings of those conversations. But instead of being able to say, "we have this appointment available" we say "come back if you're worried", or "see your family doctor if you can. | Triage Nurse 1 |
|  | You sort of need that repertoire of experience ...to have good kind of critical thinking and decision making skills because you've seen a certain you've had a certain number of reps in of seeing things. And so I think in a program where there is an it would be a big step up in responsibility. I would see it as another like layer of skill, ...maybe a bit before triage, or sort of as part of your triage kind of training and mentorship would be. You know, like, are you allowed to do the assessment that would sort of be you would be someone who would be able to offer this ED2C opportunity to families. | Triage Nurse 1 |
|  | The problem is, and I'm sure this is the same everywhere in in North America is that the nurses are really junior now, and there's a lot of turnover of staff and so we're relying on someone who's maybe had a year or not even of experience before the outer triage making these calls, and I think, as a nurse, I would find that very stressful you know, in that in that 2 min interaction to make that call where someone with like, 15 years of nursing experience. Yeah, of course, they're totally comfortable making that decision right? | Pediatric ED Physician 2 |
| Compensation | Money talks. People's time is valuable, and you have to recognize their expertise, their skill, the time that they're taking, and the time that they're taking away from other things. | Family Physician 1 |
| Resources | I would be curious to know are the same things that I can refer to in the emergency department available to me as a follow up provider to this program, because if I'm going to be seeing a [concern] that just is not connected to the resources but then I can't make a [timel] referral. Or, you know, one of these things that we can do in the emergency department. Then it's sort of like really limits me and my ability to be helpful in that situation. | Pediatrician 2 |
|  | Social work that's going to help you get the medication that you need if financial issues are a problem. You know, even things like child life is excellent...they're right there as a resource to help us out so that we can get a really good assessment and know exactly what's going on, and I just don't know if the [redirection] program would be as robust in those ways. | Triage Nurse 6 |
|  | This clinic you don't have to take on new patients you don't have to worry about follow ups... it's actually a very nice concept to just come in, do your work, leave, not have to worry about ongoing patient follow up. | Pediatrician 2 |
|  | Follow up back to that clinic. So rather than to the provider that saw them on day one if you order stuff it would be that the blood work or the X-ray or whatever gets ordered and then the doc that's in the next day would follow up on the stuff from the day before. | Family Physician 2 |
| Who | I think the having like very strict inclusion and exclusion criteria would be beneficial, so that as a registered nurse, if you were having that conversation with a parent that we're able to do it with confidence, that, you know this is a safe thing to do. | Triage Nurse 5 |
| Where | You'd want there to be some sort of arrangement in the community where you could say to someone, here, you can go in and get an X-ray and they're going to read it quickly. So whether it be an affiliation with an urgent primary care center, with X-ray capability. | Family Physician 1 |
| PED Operations | As a specialized like Children's Hospital [we have] a very like special relationship with the community where people really trust us for their pediatric care. And so, I think, being able to funnel a cohort or of patients ...to a community appointment as a way to sort of safely like decant the department when it's like super busy like that, and to also like build back trust in some of the community providers and their capacity to like manage pediatric patients in the community. I think all of that would just be super valuable. | Triage nurse 1 |
| Patient Experiences | It might also help ...shift... user perceptions of what is an emergency and what is not an emergency and ...how they're using resources. | Pediatrician 3 |
|  | Sometimes I talk to families, and they just their understanding is that they require care immediately [with information after triage] most people like the vast majority, are very happy with that, and with that understanding feel really reassured to be able to wait. | Pediatrician 2 |
|  | I feel like that would be hugely helpful in proper utilization of resources, and also people's time, because it's very stressful to be sitting in the emergency department and just worried about your child, obviously. And I feel like that would be really impactful for patients. | Pediatrician 2 |
|  | Better relationships between, you know the public and healthcare providers, too. I think when people are sitting in an emergency department for 10h it is very easy to feel overlooked and like we aren't taking the needs as seriously when it's just the lack of resources and our attention has to be focused on people who are sicker. But I think it could be really hard for people to understand, with no health background. | Triage Nurse 5 |
