# Supplementary material for: Redirecting patients from the pediatric emergency department to community locations for care: A qualitative study of healthcare professional and leader perspectives
Source: PLoS One. 2025 Dec 12;20(12):e0338725. doi: 10.1371/journal.pone.0338725 (PMC12700394; doi:10.1371/journal.pone.0338725)
Supplement: Attachment — Submitted filename: Response to Reviewers.docx [file pone.0338725.s004.docx]

To the *PLOS One* Editors,

Thank you for the opportunity to revise our research article entitled *“Redirecting patients from the pediatric emergency department to community locations for care: A qualitative study of healthcare professional and leader perspectives”* for publication in *PLOS ONE*.

We have revised this manuscript with the feedback that the academic editor and both reviewers kindly provided. Highlighted in this letter are the changes and responses to this feedback. First, we have adapted our manuscript to meet the style requirements of PLOS ONE and updated the COREQ Checklist supported information file. We’ve also reviewed the reference list in detail – a grammatical correction was made to one of the listed citations.

With respect to sharing the transcripts (i.e. the data used in this qualitative investigation), we did not receive participants consent to do so. However, we have created a supporting information file which includes iterative groupings of additional illustrative quotes (as suggested by Reviewer 1). We also updated our description about where in our data analysis process we selected illustrative quotes to present. The data availability statement in our submission form has been updated to reflect this change.

Only three positionality statements were included as three members of the research team were included in the data analysis. To clarify this, we have removed the positionality statements from the main text and included them in a table.

Finally, we feel that the sample size of our work is sufficient to accomplish our research goal – in keeping with theoretical sufficiency as a data collection endpoint. We have made comment in our limitations that relates to the potential generalizability of our research given that all participants are from the same health system.

We would like to thank the reviewers and editors again for their thoughtful review of this manuscript. In responding to the edits provided we believe that our manuscript has been strengthened and the results of our work will be meaningful for PLOS ONE readers.

Please direct any correspondence regarding this manuscript to erica.mcdonald@bcchr.ca.

Sincerely,

 Erica Qureshi, on behalf of all authors
